# Intrinsic and environmental factors in intrahepatic cholangiocarcinoma development

**DOI:** 10.18632/oncoscience.365

**Published:** 2017-10-01

**Authors:** Detian Yuan, María García-Beccaria, Mathias Heikenwalder

**Affiliations:** Department of Medical Optics, Suzhou Institute of Biomedical Engineering and Technology (SIBET), Chinese Academy of Sciences, Suzhou, China; Mathias Heikenwälder: Division of Chronic Inflammation and Cancer, German Cancer Research Center (DKFZ), Heidelberg, Germany

**Keywords:** intrahepatic cholangiocarcinoma, hepatocellular carcinoma, Kupffer cell, reactive oxygen species, tumor necrosis factor

Hepatocellular carcinoma (HCC) and intrahepatic cholangiocarcinoma (ICC) are the major primary liver cancers in adults [1]. HCC and ICC cannot be treated efficiently and have a 5 year survival upon diagnosis from ~ 30% (HCC) to ~ 5% (ICC). Consequently, liver cancer is the second most common cause for cancer related death. ICC and HCC have so far been regarded as discrete entities with distinct etiologies, genomic and transcriptomic landscapes, and prognosis, whereas phenotypic overlap and shared molecular traits may also exit [2]. Therefore, understanding the molecular mechanisms of ICC formation is essential and informative for future clinical trials.

Genetic manipulations mimicking aberrant signaling pathways or genetic mutations found in human ICC have given insights into mechanisms of ICC tumorigenesis. For example, constitutive activation of Notch signaling in hepatocytes - a well-known developmental signaling input of the biliary tree - can lead to transition into biliary cells eventually progressing to ICC [3]. Conversely, withdrawing Hippo signaling from hepatocytes that maintains their differentiated state has been shown to induce expansion of biliary/ progenitor compartment [4]. By leveraging CRISPR/ Cas9-mediated multiplex mutagenesis it was recently shown that genetic manipulation of genes predisposing to ICC/HCC together with genes commonly mutated in ICC results predominantly in ICC formation [5]. These mouse models not only support the concept that developmental and/or oncogenic signaling pathways in hepatocytes or cholangiocytes can fuel up malignant transformation, but also serve as elegant tools to elucidate uncovered mechanisms of ICC formation.

Besides the cell-autonomous mechanisms of ICC development, signaling inputs from the liver niche have been demonstrated to shape liver cell fate and the type of liver cancer [6, 7]. Since a feature of ICC- related etiologies is chronic liver damage and induction of ROS accumulation, we examined liver biopsies from ICC patients and well-established ICC mouse models - mentioned above - and observed a high prevalence of 8-OHdG+ hepatocytes surrounding ICC lesions: These ROShigh hepatocytes in ICC specimens emphasized the need for functional characterization of the oxidative liver- microenvironment during ICC formation.

**Figure 1:**
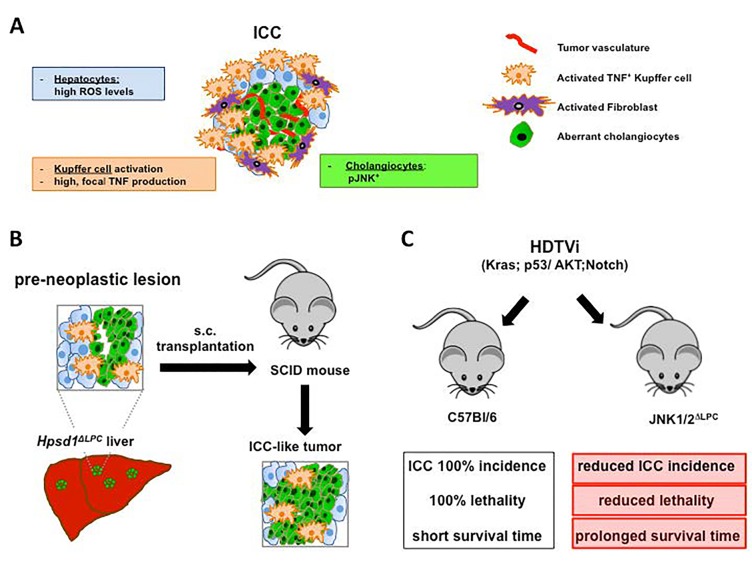
Environmental Tnf produced by Kuffper cells fuels ICC tumorigenesis (A) ROS accumulation in hepatocytes causes activation of Kupffer cells within the liver niche, resulting in focal, high Tnf production. This leads to ICC tumorigenesis by activating Jnk in cholangiocytes. (B) Subcutaneous (s.c.) transplantation of liver pieces containing premalignant lesions from Hspd1ΔLPC mice into immunocompromised mice induces ICC development. (C) Liver-specific deletion of JNK1/2 ameliorates ICC development in Akt/Notch or p53/Kras- driven ICC models, indicating an essential role of JNK1/2 in ICC development.

In this regards, we took advantage of the Hspd1flox/flox mouse line by which efficient, tissue specific mitochondrial damage-induced ROS generation can be achieved [8]. Intercrossing Hspd1flox/flox with Alb-Cre mice (Hspd1ΔLPC) resulted into liver specific deletion of Hspd1 and strong ROS accumulation [8]. Hspd1ΔLPC mice displayed a multistep carcinogenic process from hepatocyte cell death to compensatory proliferation to cholangiolar malignant transformation and the development of cholangiocellular premalignant lesions [8]. Importantly, these lesions resembled human biliary intraepithelial neoplasia (BilIN), the precursor cancerous lesion of ICC (Figure 1A). Unlike other ICC models including those mentioned above, premalignant cholangiocytes in Hspd1ΔLPC livers escaped Cre-mediated Hspd1 deletion and thus were free of high ROS accumulation, rendering this model suitable to study niche-related mechanisms of ICC tumorigenesis [3-5, 8].

Indeed, Hspd1ΔLPC livers displayed remodeling of the stromal compartment with histopathological features of human ICC, such as lymphocytes infiltration, fibroblast activation and collagen disposition, and Kupffer cell activation, which have long been recognized to contribute to the pathogenesis of ICC. Moreover, premalignant lesions resembled a malignant potential as transplantation of the latter into immunocompromised mice induced ICC (Figure 1B). Screening of ligands known to boost cholangiolar differentiation/proliferation revealed focal, extensive and rapid Tnf upregulation, coincident with robust Kupffer cell activation in the liver niche of Hspd1ΔLPC mice. Further mechanistic studies identified the Janus kinase (Jnk) as the downstream effector. Depletion of Kupffer cells with clodronate, blocking Tnf signaling by knocking-out Tnfr1, or pharmacological inhibition of Jnk diminished cholangiolar overgrowth and formation of pre-neoplastic lesions [8]. Kupffer cell-mediated Tnf production in the ICC niche and Jnk activation with aberrant cholangiocytes in ICC were confirmed in other ICC mouse models, but were absent in chronic inflammation- or NASH-induced HCC models [8]. Importantly, genetic deletion of Jnk1/2 significantly reduced ICC development in Akt/Notch or p53/Kras- driven ICC models, indicating an essential role of Jnk in ICC development [8] (Figure 1C).

We then used biopsies from patients with HCC or ICC, which we complemented with studies in distinct ICC mouse models and cultured hepatoblasts, primary cholangiocytes or HCC/ICC cell lines, to assess the involvement of ROS/Tnf/Jnk axis in ICC progression. Within human ICC niches, Tnf-expressing stroma localized close to ICC lesions with Jnk activation, forming a tumor-nourishing environment (Figure 1A). These results not only reinforce the previous clinical observation that ICC possesses a high degree of pre-cancerous stromal compartment, but also identify high, local Tnf as one of the risk factors.

Several other studies suggest conflicting roles of Kupffer cells in HCC or ICC. The role of Kupffer cells seems to be context dependent. Indeed, the cytokine signatures of Kupffer cells isolated from CDE or DDC diet models are different compared to those from ICC mouse models [6-8]. This phenomenon supports the idea that macrophages are highly adaptable to various stimuli. In addition, reciprocal education of Kupffer cells by ICC or HCC cells or the tumor microenvironment might exist, supporting the cancer-niche co-evolutionary theory of ICC/HCC and Kupffer cells.

In conclusion, our study extends growing evidence that the interplay between cancer cells, myeloid and stromal cells is a crucial determinant of cancer progression, and clearly identifies the tumor-niche as therapeutic target. For example, our current data demonstrate that Kupffer cell depletion or ROS-inhibition diminishes growth of BilINs [8]. Together with the observation that Kupffer cell ablation rewires liver progenitor cells in a toxin-mediated injury model [6], reverting a hospitable myeloid or stromal pro-carcinogenic niche may represent an attractive strategy for ICC treatment and other liver diseases characterized by dysregulated niche function.
